# Testing a multi-malaria-model ensemble against 30 years of data in the Kenyan highlands

**DOI:** 10.1186/1475-2875-13-206

**Published:** 2014-05-30

**Authors:** Daniel Ruiz, Cyrille Brun, Stephen J Connor, Judith A Omumbo, Bradfield Lyon, Madeleine C Thomson

**Affiliations:** 1 International Research Institute for Climate and Society, Lamont Doherty Earth Observatory, Columbia University in the City of New York, 61 Route 9 W, Palisades, PO Box 1000, New York 10964-8000, USA; 2 Unidad Académica Civil, Ambiental, Industrial y Geológica, Escuela de Ingeniería de Antioquia, km 02 + 200 Vía al Aeropuerto José María Córdova, Antioquia, Envigado, Colombia; 3 École Polytechnique ParisTech, 12, rue Édouard Manet, 75013 Paris, France; 4 School of Environmental Sciences, University of Liverpool, Liverpool L69 3BX, UK

**Keywords:** Malaria model ensemble, *Plasmodium falciparum* malaria, Climate change, Kenyan highlands

## Abstract

**Background:**

Multi-model ensembles could overcome challenges resulting from uncertainties in models’ initial conditions, parameterization and structural imperfections. They could also quantify in a probabilistic way uncertainties in future climatic conditions and their impacts.

**Methods:**

A four-malaria-model ensemble was implemented to assess the impact of long-term changes in climatic conditions on *Plasmodium falciparum* malaria morbidity observed in Kericho, in the highlands of Western Kenya, over the period 1979–2009. Input data included quality controlled temperature and rainfall records gathered at a nearby weather station over the historical periods 1979–2009 and 1980–2009, respectively. Simulations included models’ sensitivities to changes in sets of parameters and analysis of non-linear changes in the mean duration of host’s infectivity to vectors due to increased resistance to anti-malarial drugs.

**Results:**

The ensemble explained from 32 to 38% of the variance of the observed *P. falciparum* malaria incidence. Obtained R^2^-values were above the results achieved with individual model simulation outputs. Up to 18.6% of the variance of malaria incidence could be attributed to the +0.19 to +0.25°C per decade significant long-term linear trend in near-surface air temperatures. On top of this 18.6%, at least 6% of the variance of malaria incidence could be related to the increased resistance to anti-malarial drugs. Ensemble simulations also suggest that climatic conditions have likely been less favourable to malaria transmission in Kericho in recent years.

**Conclusions:**

Long-term changes in climatic conditions and non-linear changes in the mean duration of host’s infectivity are synergistically driving the increasing incidence of *P. falciparum* malaria in the Kenyan highlands. User-friendly, online-downloadable, open source mathematical tools, such as the one presented here, could improve decision-making processes of local and regional health authorities.

## Background

Process-based models have played a significant role in understanding the complexity of malaria transmission dynamics since the discovery of the malaria transmission pathway at the turn of the 19th century [1]. Sir Ronald Ross, while working at the Indian Medical Service in the 1890′s, demonstrated the life cycle of malaria parasites in *Anopheles* mosquitoes, and was one among the first to publish a series of papers using mathematical functions to study malaria transmission. He developed a simple model which explained the relationship between the number of mosquitoes and malaria incidence in human populations, and used it to arrive at important practical conclusions such as that, “…to counteract malaria anywhere we need not banish *Anopheles* there entirely…we need only to reduce their numbers below a certain figure.” [2] Sir Ronald Ross was also able to conclude from his modeling efforts that control programmes that integrated vector reduction (larvicides), drug treatment (quinine), and personal protection (bed nets) were much more likely to succeed than efforts that relied on just one intervention measure [2]. From a malaria policy perspective, the value of a model-based analysis of malaria transmission dependent outcomes is in the opportunity to systematically examine drivers surrounding these outcomes and their relevance to the ultimate decision being addressed.

While malaria transmission models of varying complexity have been developed over the years in response to specific needs, the basic principle of parsimony is key to model development. This principle states that among competing hypotheses, the one with the fewest assumptions should be selected. Other, more complicated solutions may ultimately prove correct, but—in the absence of certainty—the fewer assumptions that are made, the better. However, recent advances in the theory of mosquito-borne pathogen transmission seeks to better understand uncertainty in the traditional malaria modelling framework by realistically acknowledging spatial heterogeneity of transmission in complex epidemiological landscapes [3]. Another important approach to reducing uncertainty in model results is improvements in the quality and quantity of appropriate data used to both drive and test the model outputs. Access to quality controlled high spatial and temporal meteorological station data has been a particular challenge in Africa where observing stations are less than an 1/8 of the number recommended by the World Meteorological Office [4]. After identifying the most appropriate model(s) with least assumptions and using the best available data, two other sources of uncertainty must be taken into account. These are the starting conditions used to initialize the model and the specific parameterization of the model itself. For example, the seasonal evolution of malaria cases as described by a time dependant process-based model is dependent on the initial state of the gametocyte carrier rate at time t = 0. Since a perfect assessment of the gametocyte carrier rate in the population is not possible then an estimation of the most likely rate is needed to initialize the model. Choices made in model structure are also significant sources of model uncertainty.

The climate forecasting community has used multi-model ensembles to overcome challenges resulting from initial conditions and parameter and structural uncertainties in model design [5]. Ensemble approaches have been used to quantify uncertainty in future (e.g. seasonal) climate and its impacts (e.g. on malaria incidence) in a probabilistic way [6]. In this analysis disease model outputs represent a probability distribution of disease risk. In years and regions where the probability distribution is broad there will be little predictability in the system. However, where there is a sharp probability distribution, predictability will be stronger and information may be used by decision-makers for taking precautionary action. The main advantage of using a probabilistic system is that users should not be misled by overconfident erroneous forecasts in situations where predictability is small [7]. Building on the experiences of the climate forecasters, this paper describes recent advances in the effort to implement a multi-malaria-model ensemble framework and to test the validity of this approach using retrospective malaria and climate data obtained from Kericho, in the western highlands of Kenya.

Kenya’s western highlands have long been at the centre of debate over whether or not global climate change has played a significant role in the post 1980′s re-emergence and increasing incidence of *Plasmodium falciparum* malaria [8-18]. Attention has been given to reported outbreaks in, for instance, the Kisii District of Nyanza Province and the adjacent tea plantations in Kericho. Inpatient and outpatient data from these sites suggest that malaria patterns over the period 1980–2000 were characterized by increased incidence, expanded geographic areas and higher case-fatality rates [10]. Malaria-positive cases in Kericho have, however, recently declined and returned to moderate levels since 2005 [17], and such marked decline has been observed across many localities in East Africa [19]. As widely discussed in the scientific literature, changes in local climatic conditions are not the only external factor driving the observed changes in malaria epidemics [9]. Anti-malarial drug resistance [20-23], economy-driven, two-way mobility from/to endemic-prone lowlands [24], changes in mosquito populations [25], and to a lesser extent, depletion of regional health services [26], have also played a critical role in long-term changes in malaria morbidity. All these environmental, socio-economic and behavioural factors need to be considered together [27-30] in order to understand the general epidemiology of the disease and the timing and severity of *P. falciparum* malaria epidemics.

Here a multi-malaria-model ensemble framework, which comprises four well-known process-based malaria models, is implemented to assess temporal changes in malaria morbidity profiles in Kericho, in the highlands of Western Kenya. Simulations are focused on the role that long-term changes in climatic conditions (temperature and rainfall) play in driving malaria incidence, but can be expanded to the analysis of changes in non-climatic factors once related information becomes available. The potential advantages of a multi-model approach in helping decision-makers to better understand the impact of exogenous drivers of malaria risk are therefore described.

## Methods

### Study site

Analyses are focused on Tea Plantation 1 in Kericho district (1,200-3,000 m above sea level), a region of economic and political importance given its agricultural activities [23]. Kericho provides a good scenario for modelling the timing and severity of *P. falciparum* malaria outbreaks and the potential impact of changes in climatic conditions on malaria morbidity profiles. Two tea plantations in Kericho, each consisting of 18 estates, employ in average 18,000-18,500 workers whose families comprise three to four dependents each [24]. Assuming that the number of individuals has been stable over the past decades [31], the total population at risk in Plantation 1 can be assumed to reach about 27,000 individuals. Simulations presented here complement previous experiments for the Kisii District Hospital of Kisii municipality [29].

### Data

**Figure 1:**
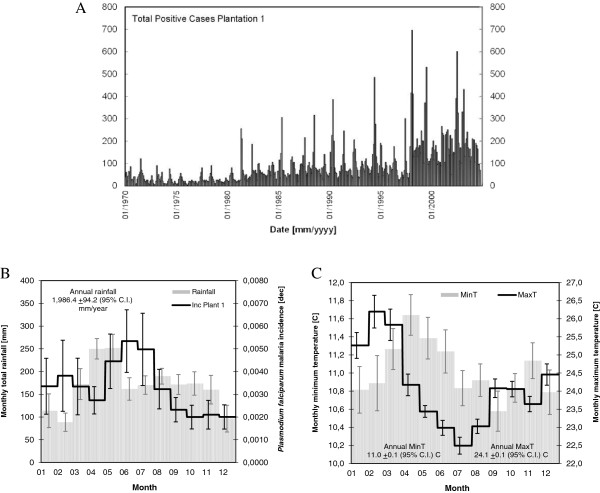
***Plasmodium falciparum *malaria and climate in Kericho District, Kenyan highlands. (A)** Historical monthly malaria positive cases observed in Tea Plantation 1 over the period spanning January, 1970 to October, 2004. **(B)** and **(C)** Annual cycles of rainfall (grey bars in panel B), minimum temperature (grey bars in panel C), and maximum temperature (black solid line in panel C) observed over the period 1979–2009. Error bars depict the confidence intervals for a 0.05 significance level. The total annual rainfall amount and its confidence interval for a 95% confidence level are presented on the top left hand side of panel B. The average minimum and maximum annual temperatures and their confidence intervals are presented on the bottom left-hand side and bottom right-hand side of panel C. See also the *P. falciparum* malaria incidence (black solid line in panel B), based on the historical monthly malaria positive cases (presented in panel A).

Data included weather station records and *P. falciparum* malaria positive cases. Quality controlled daily records of maximum temperatures, minimum temperatures and rainfall totals, gathered at Kericho meteorological station [18], located at 33°21′E and 0°21.6′S in the Kenyan highlands, were processed. Temperature records are available for the period spanning 1 January, 1979 to 31 December, 2009. Rainfall time series are available for the period spanning 1 January, 1980 to 31 December, 2009. Monthly malaria-positive cases from inpatient admission registers in Tea Plantation 1 in Kericho, spanning the period January, 1970 to October, 2004, were obtained from Figures four and six in [23], see Figure 1(A).

### Process-based models

In the multi-malaria-model ensemble proposed for this set of simulations only four mathematical tools were considered: the models proposed by Ross-Macdonald [32,33], Anderson and May [34], Worrall *et al.*[35], and Alonso *et al.*[36]. These four process-based models exemplify the ample spectrum of malaria modelling approaches: from a tool with a single dynamical, discrete equation to a process-based model with a system of 11 coupled ordinary differential equations. The Ross-Macdonald’s model (MAC model) is based upon a system of two coupled ordinary differential equations, whose dynamical variables represent the proportion of people affected and the (implicit) counterpart in the vector population. These proportions do not distinguish between infected and infectious stages. Anderson and May extended the Ross-Macdonald’s model by considering the proportions of exposed individuals and exposed mosquitoes, and by including the latency of infection in human hosts and mosquito vectors. The herein-called AM model is thus based on a system of four coupled ordinary differential equations with time lags. Worral *et al.* developed, in turn, a single discrete-equation, temperature- and rainfall-driven process-based model (WCT) to predict malaria epidemics in areas where brief seasonal transmission and occasional epidemics do not enable acquired immunity, and to examine the impact of indoor residual spraying on malaria transmission intensity. The WCT tool is composed of six submodels, which calculate the number of adult female mosquitoes feeding on human hosts, the length of the gonotrophic and sporogonic cycles, the vector survivorship in sprayed and unsprayed populations, the sporozoite rate, and the total number of new infections, superinfections and recoveries within the human population. Lastly, Alonso *et al.* developed a coupled mosquito-human model, herein called the ABP model, based upon a system of 11 coupled ordinary differential equations. In the human host component, level variables represent the susceptible non-infected human hosts, the infected but non-infectious individuals, the infected individuals who acquire asymptomatic infection but are nevertheless infectious and can transmit malaria parasites to mosquito vectors, the recovered individuals or those human hosts who have cleared parasitaemia, and the infected individuals who present symptoms and therefore receive some sort of clinical treatment. In the mosquito population, level variables depict the number of larvae, the larvae carrying capacity, and the total number of susceptible non-infected mosquitoes, infected non-infectious mosquitoes, and infectious mosquitoes. A full description of all these process-based models is presented in the Additional file 1. Their community-based, *Plasmodium* parasites, human host, *Anopheles* mosquito population and environmental parameters and exogenous variables (see Tables 1 and 2) were initially gathered from published literature.

**Table 1:** Parameters and exogenous variables – community-based, malaria parasite and human host

**Component**	**Parameter/exogenous variable**		**Process-based model**				**Note**^**%**^
			**MAC**	**AM**	**WCT**	**ABP**	
Community-based	Total human population at risk		d	d	d	N	1
	Human natural birth					B = δ_H_*N	
	Human natural mortality rate	Assuming a given average lifetime		μ_1_			1
		Individual losses due to mortality or more generally, population turnover				δ_H_	1
	Proportion of total population at risk covered with IRS program campaign				C		1
	Proportion of positive cases actually reported to health facilities				λ		1
Malaria parasite	Parasite species		*P. falciparum*				--
	Sporogony/malaria parasites incubation period		n	n = f_N_/(T + l-g_N_)	n = f_N_/(T + l*(U-υ)/U-g_N_)	γ_P_ = f(T)&	--
	Number of degree-days needed to complete parasite development			f_N_	f_N_		1
	Temperature threshold below which parasite development ceases			g_N_	g_N_		1
	Latency of infection in mosquito vectors			t_m_			2
Human host	Reciprocal of the average duration of the “affected state”		r = 1/(HD + WN)	r = 1/[HD + wn(t)]			--
	Average time in the exposed phase					1/γ	2
	Host delay for infectivity; length of the interval between infection/sporozoite inoculation and the onset of infectivity/gametocyte maturation (*HD*) or latency of infection (*t*_*h*_)		HD	t_h_			2
	External force of infection					β_e_	2
	Probability that an infectious bite results in infection					b	1
	Host window for immunity; duration of a host’s infectivity to vectors, from the first to the final present of infective gametocytes		WN	wn(t)			2
	Loss of immunity basal rate					σ_0_	2
	Human recovery	Assuming a given mean duration of infectivity			r		2
		C to S clearance rate				ρ	1
		Fraction of infections in humans that fully develops severe malaria symptoms and then receive clinical treatment				ξ	2
		Factor that decreases the per-capita transmission rate when asymptomatic but infectious individuals -I- can present a relapse of severe malaria symptoms if they are bitten again				η	2
		I to R recovery basal rate				r_0_	2
		C to I recovery rate				ν	2

& See [36]; % 1: chosen from literature and fixed constant, and 2: chosen from literature and fitted.

**Table 2:** **Parameters and exogenous variables *Anopheles *** **mosquito population and environment**

**Component**	**Parameter/exogenous variable**		**Process-based model**				**Note**^**%**^
			**MAC**	**AM**	**WCT**	**ABP**	
Mosquito population	Vector natality: rainfall-to-mosquitoes constant (μ), mosquito fecundity factor (*F*), and number of eggs per oviposition event (*n*)		μ	μ	μ	F, n	2
	Vector survivorship: daily survival probability (*p*)		p = α^(1/U)	p = α^(1/U)	p = [α*(1-C) + α*β*C]^(1/U)	〈*λ*〉 = *f*(*T*)&	
	Probability of surviving each gonotrophic cycle in an unsprayed population (not covered by the IRS campaign)		α	α	α		1
	Reduction in *α* in the population covered by the spray programme immediately after spraying				β		1
	Gonotrophic cycle		U = υ + (f_U_/(T + l-g_U_))	U = υ + (f_U_/(T + l-g_U_))	U = υ + (f_U_/(T + l-g_U_))		
	Total number of degree days needed to complete development of the ovaries		f_u_	f_u_	f_u_		1
	Minimum temperature needed to complete development of ovaries		g_u_	g_u_	g_u_		1
	Length of a part of gonotrophic cycle to find a water body and a new human host		υ	υ	υ		1
	Vector feeding		a = 0.091678*T_e_-1.7982	a = 0.091678*T_e_-1.7982	a = h/U	a = 0.091678*T_e_-1.7982	
	Human blood index (proportion of mosquitoes feeding on humans)				h		1
	Mortality rate	Assuming a given average lifespan		μ_2_			1
		Larvae mortality caused by temperature- or rain-independent processes, such as predation				δ_0_	2
		Per-capita larvae death rate -inverse of the larval average life time- at temperatures of 14, 16, 18, and 20°C				δ_L_(14), δ_L_(16), δ_L_(18), δ_L_(20)	1
		Death factor introduced to represent the washout effect for the larvae				δ_R_	2
	Vector infectivity: probability of becoming infected per infectious meal (*k*), probability of becoming infectious with malaria parasites (*v*)				*k* and *v*		1
	Proportion of *Anopheles* mosquitoes with sporozoites in their salivary glands which are actually infective		b	b			1
	Vector susceptibility or human host-to-mosquito probability of transmission			c		c	1
Environment	Daily effective temperature		T_e_ = T + (1-x_p_)*l	T_e_ = T + (1-x_p_)*l		T_e_ = T + (1-x_p_)*ΔT	
	Daily ambient temperature		T	T	T	T	
	Temperature weighting parameter		x_p_	x_p_	--	x_p_	1
	Difference between indoor and outdoor temperatures (*l*) or maximum allowed difference between the maximum temperature adult mosquitoes can experience and outdoor temperature (*ΔT*)		l	l	l	ΔT	1
	Daily/monthly rainfall		P	P	P	P and 〈*P*〉_12_&	--
	Larvae carrying capacity	Conversion factor				k_A_	2
		Loss rate				k_E_	2

& See [36]; % 1: chosen from literature and fixed constant, and 2: chosen from literature and fitted.

The following three endogenous variables of the MAC model were modified to include climate covariates: *a*, represented as a function of *T*_*e*_ following the regression between the inverse of the average gonotrophic cycle and the daily ambient temperature [36]; the anopheline density in relation to man (*m*), represented as a linear function of *μ* and the monthly rainfall [35]; and *p*, represented as a function of *U*, which in turn is dependent on the daily ambient temperature. Besides the three endogenous variables modified in the MAC model, the following two variables were changed in the AM model: *n*, represented as a function of the daily ambient temperature; and *WN*, represented as a function of time. The following four endogenous variables of the WCT model include climate covariates: the number of mosquitoes emerging each month (*q*), represented as a linear function of *μ* and the monthly rainfall; *p*, as a function of *U*; *n*, represented as a function of the daily ambient temperature; and *a*, represented as a function of the human blood index (*h*) and *U*. Lastly, the following five endogenous variables of the ABP model include climate covariates: *a*, represented as a function of *T*_*e*_; the larval mortality rate (*δ*_*L*_), as a function of the temperature-dependent larval mortality (*δ*_*L*_*(T)*) and the rainfall-dependent increase in mortality due to heavy rain (*δ*_*L*_*(P)*); the larval development rate (*d*_*L*_), as a function of the daily ambient temperature; the average lifetime of mosquitoes, (〈*λ*〉), represented as a function of the daily ambient temperature; and the *per-capita* rate at which new infectious mosquitoes arise (*γ*_*P*_), dependent on the daily ambient temperature.

### Set of simulations

Simulations proposed here included six series of experiments, which were run using the user-friendly, online-downloadable, open source computer software Scilab® 5.3. Codes developed for the analyses are available upon request. Experiments were designed to: 1) compare Scilab® 5.3 simulation outputs with analytical solutions; 2) perform simulation runs for changes in initial conditions and for seasonal variations in climatic variables; 3) simulate actual climatic conditions and assess the role of climate long-term trends, inter-annual dependency and seasonality in malaria incidence; 4) assess models’ sensitivities to changes in sets of parameters; 5) incorporate uncertainty in the predictability of malaria outbreaks; and 6) analyse the potential impact of anti-malarial drug resistance on morbidity profiles. A brief description of each of these experiments is presented below.

The first set of experiments included comparisons of simulation outputs with the results of the analytical study of equilibrium points, time to reach equilibria and time steps of the MAC and WCT models. Parameters of these two models were fixed to representative values and full certainty in their values was initially assumed.

The second set of simulations included models’ sensitivities to changes in initial conditions and simulation outputs for constant climatic conditions. As in the analysis of stability conditions, parameters of the four-malaria-model ensemble were fixed to fully certain representative values. Changes in equilibrium points and time to reach equilibria were assessed for at least five different initial proportions of infected or infectious individuals. Constant mean annual temperatures and total annual rainfall amounts, as well as historical annual cycles of mean temperature and rainfall were used to characterize local epidemiological conditions. Simulated annual cycles of malaria prevalence (once models reach their equilibria) were then compared to the historical annual cycle of *P. falciparum* malaria incidence.

The third set of experiments comprised simulations of actual climatic conditions over the retrospective period spanning January, 1979 to October, 2004, when the malaria data end. Some parameters of the four-malaria-model ensemble, which were initially set to fully certain values, were then modified to several values within a sensible range reported in the literature. In the MAC model, *HD*, *WN* and *m* were fitted using the full retrospective period January, 1979 to October, 2004. In the AM model, the following parameter values were fitted: *t*_*h*_, *WN*, *m*, and *t*_*m*_. In the WCT model, the following parameter values were modified within their reported range and later fitted: *r* and *m*. Lastly, in the ABP model the following parameters were included in this analysis: *1/g*, *b*_*e*_, *s*_*0*_, *x*, *h*, *r*_*0*_, *n*, *F*, *d*_*0*_, *d*_*R*_, *k*_*A*_, and *k*_*E*_.

Simulation outputs were compared through several statistical parameters such as the correlation coefficient (R-value) between simulated malaria cases and actual positive cases, the percentage of the variance of the actual malaria morbidity that is explained by simulation outputs (R^2^-value), the slope of the regression of simulated cases on actual cases, and the mean square and mean absolute errors. Comparisons also included a function of likelihood that is based on the probability of observing *I*_*o*_ cases given the deterministic prediction *I*, as discussed in [36]. Best set of parameters were those yielding ‘comparable predictions of actual malaria positive cases’ [36]. The ‘most likely’ models were then implemented to assess the impacts of changes in climatic conditions on *P. falciparum* malaria transmission dynamics in the highlands under study. The ensemble was run with and without long-term climatic trends, inter-annual dependency and historical seasonality, in order to address how much of a change in the size of epidemics could be attributed to changes in climatic conditions.

The third set of experiments also included multi-model simulations to the end of the Kericho temperature and rainfall data (i e, retrospective period spanning January, 1979 to December, 2009), in order to understand whether or not climatic conditions have been less favourable to malaria transmission in recent years. A full certainty in the ‘most likely’ set of parameters was also assumed in these simulation runs.

The fourth set of simulations included models’ sensitivities to changes in sets of parameters. In order to assess the impacts of changes in exogenous variables on simulation outputs of the proposed process-based models, the following discrete gradient was used to measure the models’ response to slight variations in the values of their best set of parameters, *x* =  ( *x*_1_, *x*_2_, ⋯, *x*_*i*_, *x*_*i* + 1_, ⋯, *x*_*n*_):

$$S_{i}=\frac{\frac{F\;\left(x_{1},x_{2},\cdots ,x_{i}+\Delta \;x_{i},x_{i+1},\cdots ,x_{n}\right)-F\;\left(x_{1},x_{2},\cdots ,x_{i},x_{i+1},\cdots ,x_{n}\right)}{F\;\left(x_{1},x_{2},\cdots ,x_{i},x_{i+1},\cdots ,x_{n}\right)}}{\frac{\Delta x_{i}}{x_{i}}},$$

where *F* ( *x*_1_, *x*_2_, ⋯, *x*_*i*_, *x*_*i* + 1_, ⋯, *x*_*n*_) denotes the simulation outputs function for all the parameters. Also, the Sobol Index was used to assess the sensitivity of a given model to slight changes in its set of parameters. For the WCT model, for instance, *m*, the proportion of mosquitoes feeding on humans (*h*), *l*, *a*, *k*, *v*, *u*, *r*, *f*_*u*_, *g*_*u*_, *f*_*N*_, *g*_*N*_, and the proportion of humans that are infectious (*x*) were all included in the analysis of WCT sensitivity.

The fifth set of simulations explored the role of uncertainty in the predictability of malaria outbreaks. Numerical simulations generated distributions of monthly cases or *P. falciparum* malaria prevalence by taking into account uncertainty in parameter values (i e, introducing parameter ranges in simulation runs). Twenty-five, 50 and 95% percentiles of the distributions of simulated primary cases or malaria prevalence were plotted for each month and compared to actual positive cases or *P. falciparum* malaria incidence. Simulations also included time lags of zero, one and two months.

The sixth and last set of simulations focused on the analysis of non-linear changes in the mean duration of host’s infectivity to vectors, from the first to the final present of infective gametocytes, due to increased resistance to anti-malarial drugs [20,37] and the influence of higher transmission on its spread [38]. Although chloroquine resistance was first reported in Kenya in the late 1970s [39], only by 1996 were clear signs of increased resistance reported in Kericho [20]. The recovery rate was thus set to reflect high sensitivity of malaria parasites to chloroquine in the mid-1980s, and low to moderate sensitivity by the mid- to late-1990s. The proposed non-linear fashion allows representing that approximately half of clinical infections did not clear thoroughly by the end of the available historical period [20]. A single simulation run was compared with the 25, 50 and 95% percentiles of the distributions of monthly *P. falciparum* malaria prevalence suggested by the multi-malaria-model ensemble for time lags with the highest R^2^-values.

### Analysis of climate data

Annual cycles of observed rainfall and minimum and maximum temperatures were calculated and compared with the annual cycle of *P. falciparum* malaria incidence. Annual values of several climatic variables were then computed. Climate variables included the diurnal temperature range, which has been suggested to be important in the analysis of malaria transmission dynamics [14]. Total December-January-February, March-April-May, June-July-August, and September-October-November rainfall amounts, dry days and maximum dry spells were also processed to support the analyses. A total number of 24 climatic variables were analysed: 12 for rainfall, four for minimum temperature, four for maximum temperature, and four for the diurnal temperature range.

Long-term linear trends in observed and simulated annual time series were identified using simple regression analysis, and trend magnitudes were calculated by the method of least squares. Upper and lower confidence limits were also computed for the simple linear regression models. Confirmatory analyses were implemented to assess the statistical significance of the observed trends. Four hypothesis tests: the Student’s t-test, the Hotelling-Pabst test, the non-parametric Mann-Kendall test [40], and the aligned rank Sen’s t-test [41], were all used to assess the null hypothesis of statistically significant (at a α = 0.05) linear trends in annual time series. Serially independent yearly time series were assumed when implementing the non-parametric Mann-Kendall test. A historical time series was considered to have a statistically significant trend at a α = 0.05 significance level when at least three of the implemented hypothesis tests accepted the null hypothesis of a trend in the mean.

Wavelet analysis [42,43] was conducted to assess the dominant periodic signals in observed monthly time series of minimum temperature, maximum temperature and total rainfall. Monthly one-dimensional series were decomposed into two-dimensional time-frequency space using wavelet plots. Seasonality, interannual variability associated with the El Niño-Southern Oscillation (ENSO), and longer interdecadal fluctuations were studied in global wavelet plots. Long-term linear trends and dominant periodic signals were then removed from historical time series to compare, in anomalies plots, actual malaria morbidity profiles with simulated malaria incidence.

## Results

### Climate and malaria

Rainfall amounts observed in Kericho over the period 1979–2009 exhibit a seasonal cycle that fits the long rains and short rains climatology expected for Western Kenya, see Figure 1(B). The highest peak commonly occurs during the months of April and May, whose monthly values reach about 250–260 mm. A dry season usually takes place during the quarter December-January-February with rainfall amounts ranging from 90 to 115 mm/month. Minimum temperatures exhibit a bimodal annual cycle with peaks of 11.6°C and 11.1°C occurring during the months of April and November, respectively, and historical low values of about 10.6°C usually taking place in September, see Figure 1(C). Maximum temperatures show an annual cycle with a peak in February of about 26.2°C and a minimum value of 22°C in July, see Figure 1(C). Mean temperatures exhibit, in turn, a seasonal distribution with a peak in the months of February and March of about 18.5°C and a minimum value in July of 16.7°C. The dominant periodic signals in the historical monthly time series of rainfall are six months, 12 months and 32 to 64 months; the remaining signals are beyond the cone of influence in the global wavelet power spectra. The dominant signals in the historical monthly time series of minimum and maximum temperatures are six months, 12 months, 40 to 48 months, and 64 to 96 months; the latter, however, is also beyond the cone of influence. The dominant interannual variability could therefore be represented by a 3.4-year period sinusoid.

*Plasmodium falciparum* malaria incidence observed in Tea Plantation 1 exhibits, in turn, a bimodal annual cycle with peaks in the months of February and June-July of about 3.8 and 5.3-5.0 positive cases per 1,000 inhabitants, see Figure 1(B). Minimum malaria incidence is commonly observed during the months of October-November-December with values reaching 2.0 positive cases per 1,000 inhabitants. The June-July peak in malaria incidence follows the maximum monthly rainfall with a two-month time delay. It also shows to follow the peak in mean temperatures with a four-month timelag.

Additional file 2 presents the historical values of the set of observed climatic variables under study and the long-term trends in their annual time series. The historical annual rainfall (R1) reaches 1,986 mm/year with a 95% confidence interval of ±94.2 mm. Only the total number of dry days per year (R2) exhibited a statistically significant (at α = 0.05) long-term linear trend of about +7.4 days per decade. Historical annual average minimum (ATmin) and maximum (ATmax) temperatures reach 11.0 ± 0.1°C (95%) and 24.1 ± 0.1°C (95%), respectively. Minimum temperatures on the warmest days (MTmin), annual minimum temperatures (ATmin) and day-to-day standard deviation of minimum temperatures (SDTmin) showed increasing trends of +0.4, +0.2 and +0.1°C per decade, respectively. Maximum temperatures on the warmest days (MTmax), annual maximum temperatures (ATmax) and maximum temperatures on the coldest days (mTmax2) exhibited increasing trends of +0.2, +0.3 and +0.2°C per decade, respectively. The rest of the annual historical time series did not show statistically significant trends at a = 0.05. Mean annual temperatures thus likely increased at a rate of +0.25°C per decade over the period 1979–2009. This rate of change is consistent with trends reported by previous studies [18].

### Simulation outputs

Additional file 3 depicts the MAC, AM, WCT, and ABP simulation outputs for the historical annual cycles of mean temperature and rainfall. For the set of parameters defined in the analysis of base scenarios, models overestimate the historical *P. falciparum* malaria incidence in 0.7 to 4.0 positive cases per 1,000 inhabitants. They also exhibit, on average, a unimodal annual cycle with a peak in the months of May and June, compared to the observed bimodal seasonal distribution of malaria incidence. Moreover, process-based models show different abilities to fit the baseline seasonality that are likely to come from the way they describe different aspects of the *P. falciparum* malaria transmission cycle. The MAC, AM and WCT models are driven by the combined effects of mean temperature and rainfall, whereas the ABP model is influenced by the dynamics of the force of infection and its two main components (the local transmission and the external force of infection), as well as by the fluctuations of the larvae carrying capacity, which in turn are controlled by rainfall. Additional file 3 also displays the WCT results for various mean durations of infectivity. Changes in the infectivity from 40 to 95 days (equivalent to changes in the human host recovery probability from 0.7500 to 0.3158 month^−1^) increase simulated *P. falciparum* malaria prevalence from 2.3 to 5.2 positive cases per 1,000 inhabitants in the months of September and October, and from 5.7 to 13.3 positive cases in March; i e, changes in the mean duration of infectivity have a strong impact on malaria prevalence particularly after the February and March peak of mean temperatures.

For full certainty in its best set of parameters and for the actual climatic conditions observed over the full retrospective period 1979–2009, the four-malaria-model ensemble explains approximately 33% of the variance of monthly *P. falciparum* malaria incidence in Kericho, with a mean square error of about 1E-05. Individual simulation outputs explain from 20 to 30% of the variance, and thus are below the R^2^-value obtained by the four-malaria-model ensemble. For +0.15, +0.25 and +0.35°C per decade detrended time series, the total variance explained decreases from 33 to 24.3%, 14.4 and 4.0%, respectively. The mean square error remains constant. When the +0.25°C/decade long-term trend and the 3.4-year cycle are removed from the climatic time series, individual MAC, AM, WCT, and ABP simulation outputs show different results. R^2^-values of the MAC and AM models suggest that almost all the correlation between simulated malaria prevalence and actual malaria incidence is explained by the long-term trend and the interannual dependency. ABP and WCT simulation outputs indicate that the seasonal cycle explains most of the variance of the observed *P. falciparum* malaria incidence. Lastly, for the actual climatic conditions observed over the period 2005–2009, ensemble simulation outputs suggest that *P. falciparum* malaria prevalence reduced from 13.8 positive cases per 1,000 inhabitants to almost 5.1 primary cases over the last five years of the retrospective period. Simulation runs thus suggest that climatic conditions have likely been less favourable to malaria transmission in the area under study in recent years.

**Figure 2:**
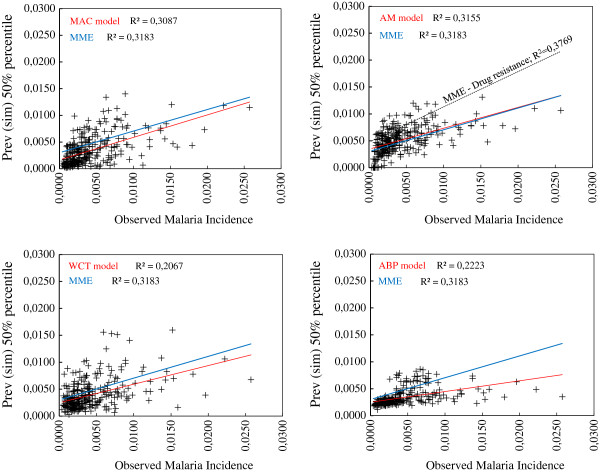
**Malaria-model ensemble simulation outputs.** Monthly *P. falciparum* malaria incidence observed in Kericho over the period spanning January, 1979 to October, 2004 (x-axes) *versus* the 50% percentile of the distributions of monthly *P. falciparum* malaria prevalence (y-axes) simulated by the MAC (upper left panel), AM (upper right), WCT (lower left), and ABP (lower right) models, for the actual climatic conditions, for the period spanning January, 1979 to December, 2009, and for 1-, 1-, 2-, and 0-month time lags, respectively. Red and blue solid lines represent the adjusted linear trends (see R^2^-values on each panel) for each model and for the four-malaria-model ensemble (MME), respectively. Dashed black line in the upper-right panel depicts the adjusted linear trend for the MME when non-linear changes in the mean duration of host’s infectivity to vectors are considered.

Additional file 4 shows the 25, 50 and 95% percentiles of the distributions of monthly *P. falciparum* malaria prevalence simulated by the MAC, AM, WCT, and ABP models, for actual climatic conditions, and for one-, one-, two-, and zero-month time lags, respectively. These lags exhibited the highest correlation coefficients between observed malaria incidence and simulated prevalence. Simulation outputs for uncertainty in parameter values included, respectively, 90, 142, 131, and 131 runs of these models (a grand total of 494 set of parameters were simulated). R^2^-values of the 50% percentiles reached 30.9, 31.6, 20.7, and 22.2%, respectively, as presented in the scatter plots in Figure 2. The highest R^2^-values of the MAC and AM 50% percentiles (35.7 and 32.7%, respectively) were obtained in the quarter December-January-February (DJF), suggesting that these models can capture the February peak in the historical bimodal annual cycle of *P. falciparum* malaria. The highest R^2^-values of the WCT and ABP models (26.9 and 46.3%, respectively) were obtained in the trimesters March-April-May (MAM) and September-October-November, indicating that these models most likely represent the periods of minimum malaria incidence.

**Figure 3:**
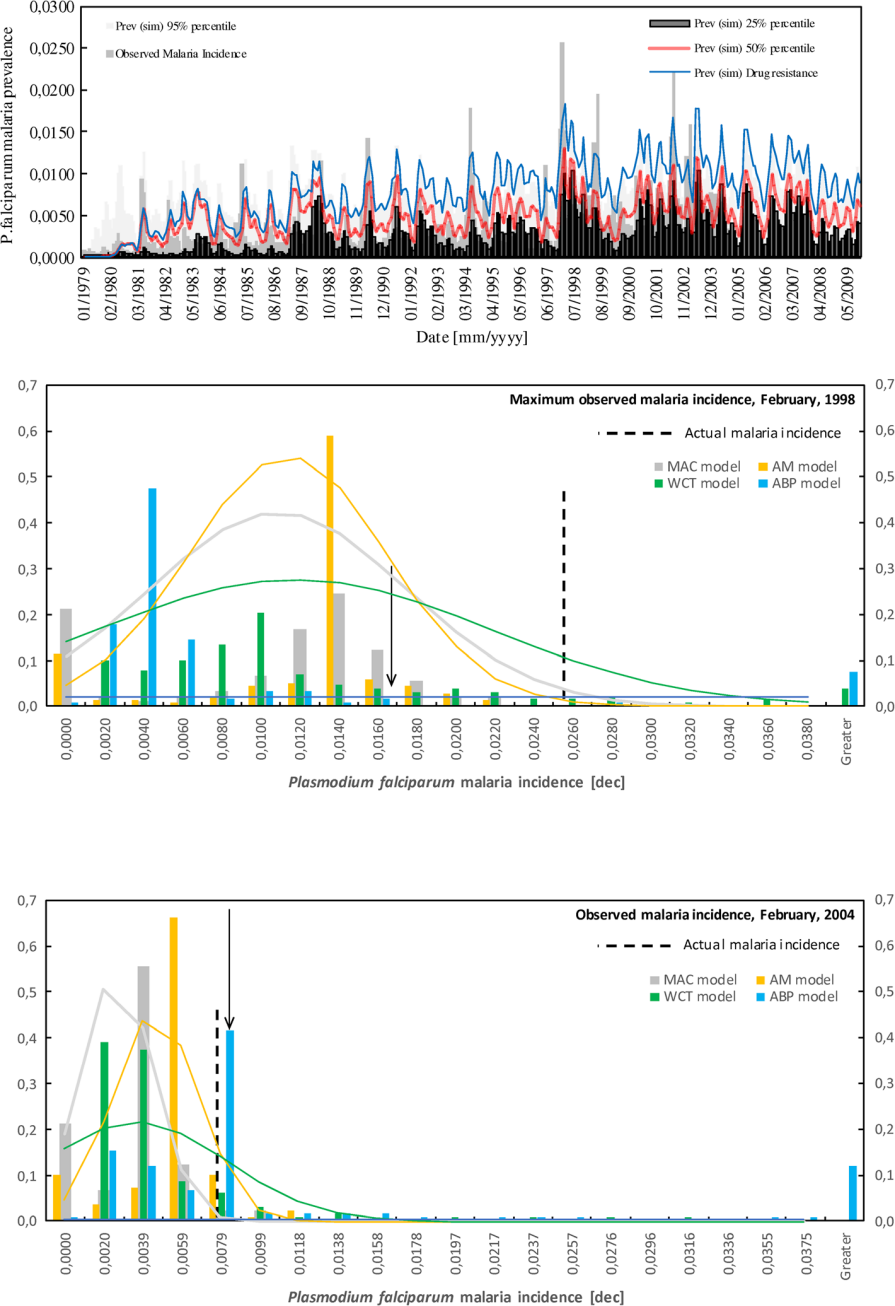
**Uncertainty in multi-malaria-model ensemble simulation outputs.** (Upper panel) Monthly *Plasmodium falciparum* malaria incidence observed in Kericho for the period spanning January, 1979 to October, 2004 (grey solid bars), along with the 25, 50 and 95% percentiles of the distributions of monthly *P. falciparum* malaria prevalence suggested by the multi-model ensemble for the actual climatic conditions and for the period spanning January, 1979 to December, 2009. Simulations of the MAC, AM, WCT, and ABP models include 1-, 1-, 2-, and 0-month time lags, respectively. See also the monthly *P. falciparum* malaria prevalence theoretically suggested by the four-malaria-model ensemble for non-linear changes in the mean duration of host’s infectivity to vectors (blue solid line). (Lower panels) Spread of individual model outputs for two specific malaria outbreaks: February, 1998 (maximum observed malaria incidence, middle panel) and February, 2004 (lower panel). Frequency histograms – frequency of MAC, AM, WCT, and ABP simulation outputs (see y-axes) in each malaria incidence interval class (see x-axes)– are depicted by colored bars. Colored lines represent the continuous probability distributions of MAC, AM, WCT, and ABP simulation outputs for each month. Vertical dashed lines depict the actual *P. falciparum* malaria incidences in each month. Vertical arrows show the theoretical *P. falciparum* malaria prevalence suggested by the four-malaria-model ensemble for non-linear changes in the mean duration of host’s infectivity to vectors.

Lastly, Figure 3 shows the 25, 50 and 95% percentiles of the distributions of monthly *P. falciparum* malaria prevalence simulated by the four-malaria-model ensemble. 31.8% of the variance of *P. falciparum* malaria incidence is explained by the ensemble median. However, if individual simulation outputs are merged at a monthly timescale, the ensemble median explains 36.7% of the variance of the observed *P. falciparum* malaria. Also, ensemble simulation runs showed their highest R^2^-values of 32.5 and 31.2% in the quarters DJF and MAM, respectively. Figure 3 also depicts the monthly *P. falciparum* malaria prevalence suggested by the ensemble for non-linear changes in the mean duration of host’s infectivity to vectors. In this case, 37.7% of the variance of malaria incidence is explained by simulation outputs. Moreover, Figure 3 shows the spread of individual model outputs for two specific malaria outbreaks. Frequency histograms and continuous probability distributions show different predictability levels in individual simulation outputs.

## Discussion

This paper described the results of the implementation of a multi-malaria-model ensemble framework to assess temporal changes in malaria morbidity profiles in Kericho, in the highlands of Western Kenya. Since the ensemble framework merges process-based models that are highly sensitive to changes in the duration of the sporogonic cycle, the gonotrophic cycle, and the survival probability of the mosquito vector, which are strongly affected by ambient temperatures, the tool mostly allows the assessment of the impacts of changes in climatic conditions on malaria morbidity profiles. In the foreseeable future the multi-model ensemble can be, however, easily expanded to assess the role of changes in non-climatic factors.

Malaria, as many vector-borne diseases, is highly sensitive to even small variations in ambient temperatures. Previous studies [10,12,18,30,44-47] suggest that changes in climatic conditions cannot be ruled out as potential drivers of the observed increases in *P. falciparum* malaria in the highlands of Western Kenya. Ensemble simulation runs presented here suggest that from 8.7 to 18.6% of the variance of *P. falciparum* malaria incidence observed in the site under study over the period 1979–2004 could be attributed to the +0.19 to +0.25°C per decade statistically significant long-term linear trend in near-surface air temperatures that took place over the period 1950–2009. Ensemble simulation outputs also suggest that climatic conditions have likely been less favourable to malaria transmission in Kericho in recent years.

Even though the four-malaria-model ensemble overestimates the historical *P. falciparum* malaria incidence when the annual cycles of mean temperature and rainfall are assumed in base scenario experiments, simulation outputs for actual climatic conditions (assuming certainty and uncertainty in parameter values) observed over the selected retrospective period do not fully capture the magnitude of the peaks in malaria incidence. Simulation runs indicate that on top of the aforementioned 8.7 to 18.6% increase in the variance of *P. falciparum* malaria incidence that could be attributed to the long-term trend in ambient temperatures, at least 6% of such variability or over ten positive cases per 1,000 inhabitants during recent peaks in the incidence could be related to the increased resistance to anti-malarial drugs. Hence, long-term changes in climatic conditions and non-linear changes in the mean duration of host’s infectivity could be synergistically driving the increasing incidence of *P. falciparum* malaria in the highlands of Western Kenya.

Which models should be considered in the multi-malaria-model ensemble? Intuitively, those models that better represent the most relevant aspects of the *P. falciparum* malaria transmission cycle, and that exhibit high accuracy and predictive power should be picked. From an operational point of view, it should be preferred to include those models that show a high skill level using a short list of parameters and exogenous variables, which can be easily measured in the field or under controlled laboratory conditions. In addition, models that are consistent with other related tools, that are not complicated, and that in the general sense are useful for routine activities of health services should be chosen. How should the results of the best malaria transmission models be combined? Simulation runs in this set of experiments were combined using equally weighted models. In theory, models with higher reliability and consistency should weigh more than those with lower skill level [48]. Future work will therefore address the need to consider R^2^-values between simulated malaria cases and actual positive cases, mean square errors, a function of likelihood, or the ‘bias’ and ‘convergence’ criteria [49] for deriving differential model weighting.

There are various limitations in the use of a malaria process-based multi-model ensemble. Models usually describe different aspects of the transmission cycle of *P. falciparum* malaria. As a consequence, some process-based models are driven by ambient temperature while others are strongly influenced by rainfall. Hence, there is a need to initially judge, subjectively and based on pure expertise, which model is suitable for a specific application. Also, simulation experiments cannot span the full range of possible combinations of parameter values and initial conditions due to time and computational capacity constraints. That is why the fine-tuning process of model parameters involves purely subjective judgment, making it hard to guarantee the proper identification of the ‘optimum location’ in the parameter space [48].

## Conclusions

Malaria control specialists need simple, open source tools such as the ones discussed here to make better decisions regarding malaria control investments, particularly now that the impact of current and future climate is increasingly considered important in the development of malaria control and evaluation strategies. Instead of using individual process-based models in isolation, however, authorities may gain more useful insights by developing ‘ensembles’ of different models, where biases in one tool may be compensated by biases in other models. The approach presented here is to use different sets of parameter values for each model and for all the proposed process-based models, and present combined simulation outputs as probability distributions. These experiments are robust in the sense that each process-based model has been subjected to several control simulations, including base scenarios and stability conditions, as well as multiple sets of runs for different choices of parameter values. As mentioned above, results suggest that the mean and the median of the malaria-model ensemble outputs outperformed individual model simulation runs. Results also incorporated the level of uncertainty associated with modelling outputs.

## Competing interests

The authors declare that they have no competing interests.

## Authors’ contributions

DR processed, analysed and interpreted weather station data, implemented the hypothesis tests for the analysis of homogeneity, coded the malaria process-based models, proposed and performed the set of simulations, carried out the comparisons between simulated malaria cases and actual positive cases, and drafted the manuscript. CB coded the malaria process-based models, helped to perform the simulations and carried out the comparisons. JAO and BL participated in the design of the study and processed, analysed and interpreted weather station data. SJC and MCT conceived and designed research activities, revised the manuscript and gave final approval of its final version. All authors read and approved the final manuscript.

## Supplementary Material

Additional file 1 Process-based modelling of malaria transmission dynamics. Click here for file

Additional file 2 Historical values and long-term trends in observed weather data. Click here for file

Additional file 3 Simulation outputs for base scenarios. Click here for file

Additional file 4 **Individual model simulation outputs **[50-66]**.** Click here for file
