# Supplementary material for: Testing a multi-malaria-model ensemble against 30 years of data in the Kenyan highlands
Source: Malar J. 2014 May 30;13:206. doi: 10.1186/1475-2875-13-206 (PMC4090176; doi:10.1186/1475-2875-13-206)
Supplement: Additional file 1 — Process-based modelling of malaria transmission dynamics. [file 1475-2875-13-206-S1.docx]

**Process-based modelling of malaria transmission dynamics**

**The Ross-Macdonald’s model (MAC)**

The Ross-Macdonald’s model is expressed by the following system of two differential equations:

$\frac{dX}{dt}=a b m Y-X \left(a b m Y+ r\right)$, and

$\frac{dY}{dt}=a X-Y \left[ a X- \ln\left(p\right) \right]$,

where the dynamical variable X represents, according to Macdonald, ‘the proportion of people affected’, and the dynamical variable Y its (implicit) counterpart in the vector population. Parameter m denotes the anopheline density in relation to man, a the average number of men bitten by one mosquito in one night, b the proportion of those anophelines with sporozoites in their salivary glands which are actually infective, p the probability of a mosquito surviving through one whole day, and r the proportion of affected people, who have received one infective inoculum only, who revert to the unaffected state in one day. The parameter r is reciprocal of the average duration of the ''affected state'', and is roughly equivalent to $\frac{1}{HD+WN}$, where HD denotes the host delay (length of the interval between infection or *sporozoite* inoculation, and the onset of infectivity or gametocyte maturation in a host) and WN represents the host window (duration of a host’s infectivity to vectors, from the first to the final presence of infective gametocytes).

The crucial aspects of the Ross-Macdonald’s model are summarized in the formula for Z_o_, the Basic Reproduction Rate of malaria:

,

where the parameter n represents ‘the time taken for completion of the extrinsic cycle’, and:

,

summarizes the ‘Vectorial Capacity’ of malaria.

Macdonald derived Z_o_ as an estimate of the average number of secondary cases arising in a very large population at risk of completely susceptible humans following the introduction of a single primary case. Z_o_=1 was defined as the transmission threshold: for values above, malaria cases will propagate; for values below, the disease will recede. The formula for Z_o_ holds that the influence of vector survivorship (p) is greater than the influence of the average number of men bitten by one mosquito in one day (a) or the sporogonic cycle (n), which are in turn greater than the influence of the proportion b, the density m, or the proportion r. Hence, Macdonald considered the vector survivorship as the single most important element in the Basic Reproduction Rate of malaria.

Macdonald’s affected proportions do not distinguish between infected and infectious stages. His conclusion with respect to host infectivity was: ‘transmission can be altered by reduction of the mean period of infectivity of a case of malaria. The influence is, however, relatively low; the reproduction rate varies directly with the mean duration of infectivity, very great changes in which would be necessary to reduce the high rates common in Africa and some other places below the critical level’. Macdonald’s b is a measure of incidence (e.g. by its role in expressions for ‘inoculation rate’ and ‘force of infection’), and r the reciprocal of the average duration of the ‘affected’ state. Macdonald wrote that ‘in nature, the value of the reproduction rate is greatly influenced by immunity altering the values of r and b’.

The stock-flow model of the Ross-Macdonald’s model is shown in Figure 1.

**The Anderson and May’s model (AM)**

Anderson and May extended the Ross-Macdonald’s model by considering the proportions of exposed individuals (E_h_) and exposed mosquitoes (E_m_), and by including the latency of infection in human hosts (t_h_) and mosquito vectors (t_m_). The AM model is thus based on the following system of four coupled ordinary differential equations (note time lags in the equations):

,

,

, and

.

Variable a denotes the mosquito biting rate. Variable m, or mosquito density, is calculated through the linear regression , where μ_3_ represents the rainfall to mosquito constant, R the total monthly rainfall, and d the total human population at risk. Lastly, parameters μ_1_ and μ_2_ represent, respectively, the mortality rates of humans and mosquitoes. μ_2_ is given by , where p, the daily survival probability of the mosquito vector, is expressed as , and U denotes the total gonotrophic cycle length.

**The mathematical model proposed by Worrall, Connor and Thomson (WCT)**

This temperature- and rainfall-driven dynamic model of malaria transmission was proposed to predict epidemics in areas where brief seasonal transmission and occasional epidemics do not enable acquired immunity, and to examine the relationship between the intervention timing and transmission intensity on the effectiveness of indoor residual spraying (IRS). WCT is composed of six sub-models that allow estimating:

- (SM1) the number of adult female mosquitoes as a function of rainfall,
- (SM2) the length of the gonotrophic or feeding cycle as a function of temperature,
- (SM3) the duration of the sporogonic cycle as a function of temperature,
- (SM4) the vector survivorship in terms of survival probability per gonotrophic cycle and per day,
- (SM5) the sporozoite rate, and
- (SM6) the number of new infections, super-infections and recoveries.

The combination of SM3 and SM4 allows calculating ‘the probability of the vector surviving long enough for sporogonic development to be completed’. SM4 is also used ‘to simulate the effects of a residual spray program, which is considered in terms of its impact upon the probability of vector survival per gonotrophic cycle’.

In SM1, the number of mosquitoes emerging each month (q) is estimated through the linear regression $q=\mu R$, where μ represents the linear scaling factor (or rainfall to mosquito constant) and R the monthly rainfall total. In SM2, the total gonotrophic cycle length (U) is estimated as:

,

where (or u) represents the period of time required by a female *Anopheles* to digest the blood meal (maturation of the ovaries), and υ denotes the length of the period required by the adult mosquito to search for a suitable water body, lay the cohort of eggs, find a new host and bite again. f_u_ represents the total number of degree-days needed to complete development, g_u_ the threshold temperature below which development ceases, and (T+l) the indoor temperatures, which are function of the outdoor ambient temperatures (T) and an adjustment factor (l). In SM3, the length of the sporogonic cycle is expressed as:

,

where f_N_ represents the number of degree-days needed to complete development, and g_N_ denotes the temperature threshold below which development ceases. Temperature T_N_ is ‘adjusted to account for differences between indoor and outdoor resting temperatures, using a weighting system, based on the period of time the vector spends indoors as a proportion of gonotrophic cycle length’, .

SM4 defines two populations of mosquitoes: covered (C) and not covered (1-C) by the spray program, where C represents the percentage coverage achieved by the spraying campaign. The daily survival probability of the mosquito vector is expressed as , where α denotes the probability of the vector surviving each gonotrophic cycle (assumed to be constant) in the population not covered by the campaign. α is reduced by β in the population covered by the spray program immediately after spraying, gradually increasing back towards α at a rate of β/6 per month over the effective residual life of the insecticide (assumed to be 6 months). Thus, the mean probability of the daily survival (P) for the whole mosquito population is given by:

.

In SM5, the sporozoite rate (S) is estimated using the equation: , where x denotes the proportion of humans that are infectious, h the proportion of human blood fed mosquitoes, k the probability of the vector becoming infected per infectious meal, and v the probability of the vector becoming infectious with the malaria parasite. The formula uses the probability of surviving the gonotrophic cycle for an unsprayed population (α); in a sprayed situation this parameter is substituted for $\left(\alpha(1-C)+\alpha\beta C\right)$.

Lastly, in SM6 the frequency at which mosquitoes feed on humans (a) is estimated as h/U, and the number of infectious mosquitoes biting humans is calculated as the product of the sporozoite rate (S), the number of mosquitoes (q), and the person biting habit (a). The probability of a human receiving an infectious bite (R) is given by , where d denotes the total human population at risk. The number of people recovering at time t (c_t_) is given by $c=\left(I-Z\right) r$, where I represents the number of infected humans, r the probability of recovery of new infected humans after time t, and Z the number of super-infections (or Z= R I). Thus, the number of infected humans at time t (I_t_) is given by:

,

where F_t_ denotes the number of people newly infected at time t or R(d-I). The total number of reported infected humans at time t is given by: λ*I_t_, where λ denotes the proportion of positive cases in the community reported to health facilities.

The schematic diagram of the WCT model and the stock-flow diagrams of its SM3 (estimation of the length of the sporogonic cycle) and SM6 (dynamics of new infections, super-infections and recoveries) sub-models are depicted in Figure 2.

**The coupled mosquito-human model of malaria proposed by Alonso, Bouma and Pascual (ABP)**

The ABP coupled mosquito-human model was implemented to assess the impacts of increasing trends in local ambient temperatures on malaria transmission dynamics in a highland region of East Africa. The model was run with and without temperature trends over a three-decade simulation period in order to address how much of a change in the size of epidemics could be attributed to the observed warmer temperatures. The model couples the dynamics of the disease in two main components: the human population and the mosquito vector. The former consists of a classical system of ordinary differential equations in which five different classes are considered: susceptible non-infected human hosts (S), infected but non-infectious individuals (E), infected individuals who acquire asymptomatic infection but are nevertheless infectious and can transmit malaria parasites to mosquito vectors (I), recovered individuals/acquired immunity or those human hosts who have cleared parasitaemia, or have too low a level of parasitaemia to effectively infect the vector (R), and infected individuals who present symptoms and therefore receive some sort of clinical treatment (C). The system of ordinary differential equations for this component is given by:

,

,

,

, and

.

In the formulation of the full version of the ABP model, the authors do not include the parameters Ψ and α in the dynamics of the total number of infected individuals who acquire asymptomatic infection (I) and the total number of infected individuals who present symptoms and therefore receive clinical treatment (C), respectively. Thus, the right-hand side of their equations only includes five terms.

Exogenous variables B and δ_H_ in the system of equations for the human host component represent, respectively, the replenishment of susceptible individuals through immigration or birth, and the individual losses due to mortality or more generally, population turnover. In the model, variables B and δ_H_ balance each other so that the total human population at risk (N=S+E+I+R+C) remains constant. Thus, B=δ_H_N.

The transmission rate, β, or “force of infection”, or the per-capita probability that susceptible individuals acquire the infection per unit time as a result of infecting bites from mosquitoes, is given by:

,

where b is the probability that an infectious bite results in infection, M(t) the total population of mosquitoes, ω(t) the fraction of infectious mosquitoes at time t, and a the number of bites per individual mosquito per unit time. The first term in β represents the local transmission where the fraction of infectious mosquitoes at time t is a consequence of acquiring the parasite from infectious humans at some earlier time. The second, additive term β_e_ is an external force of infection and represents, for example, those individuals who acquire malaria infection when visiting endemic areas.

The variable σ represents the rate of loss of immunity and is a function of the intensity of mosquito exposure (as the frequency of mosquito bites increases, the rate of loss of immunity tends to decrease) as follows:

,

where Λ denotes the rate of infectious bites per human, which is given by $\Lambda=\frac{a W}{N}$, and σ_0_ the loss of immunity basal rate, i.e. when Λ tends to zero. The variable W represents the total number of infectious mosquitoes. The variable ρ represents the C→S clearance or recovery rate. The variable r represents the I→R clearance or recovery rate and is a function of the intensity of mosquito exposure (as the frequency of mosquito bites increases, the recovery rate tends to decrease) as follows:

,

where r_0_ denotes the I→R recovery basal rate, i.e. when Λ tends to zero.

$\frac{1}{\gamma}$ denotes the average time in the exposed phase. ξ represents the fraction of infections in humans that fully develops severe malaria symptoms and, then, receive clinical treatment (C). η denotes a factor that decreases the per-capita transmission rate (β) when asymptomatic but infectious individuals (I) can present a relapse of severe malaria symptoms if they are bitten again. Lastly, variable ν represents the C→I recovery rate.

Figure 3 depicts the stock-flow model of the human host component of the ABP coupled mosquito-human model. The mosquito population model considers, in turn, the number of larvae (L) and adult mosquitoes (M); see Figure 4. The dynamics of the level variable L is given by:

,

where f is the per-capita fecundity rate or the maximum number of eggs that enter the larval stage per female per unit time. Since female mosquitoes lay eggs only once between blood meals, the fecundity and biting rates can be related following f = F a, where F denotes the mosquito fecundity factor.

The number of eggs per adult female and unit time can be written as: , where n is the number of eggs per oviposition event. The per-capita fecundity rate, f, should be lower than N_f_ to account for some mortality at the egg stage.

The variable K denotes the larvae carrying capacity and is determined by rainfall, since water pools of different size provide the habitat for mosquito breeding and larvae development. Also, larvae survival decreases when precipitation is large, above a given threshold that depends on how much rain has fallen in previous months. The carrying capacity is modelled in ABP following:

,

where k_A_ and k_E_ represent, respectively, the carrying capacity conversion factor and loss rate. Variable δ_L_ denotes the larval mortality rate and is approximated as follows:

,

where δ_0_ is a basic mortality rate caused by temperature- or rain-independent processes, such as predation. The temperature-dependent larval mortality rate, δ_L_(T), is given by:

,

where δ_L_(14), δ_L_(16), δ_L_(18), and δ_L_(20) denote the per-capita larvae death rate (inverse of the larval average life time) at temperatures of 14, 16, 18, and 20°C.

δ_L_(P) is a function that depends on daily rainfall and measures how much mortality rate increases as a consequence of heavy rain. Specifically, δ_L_(P) is given by:

,

where δ_R_ is a proportional factor transforming a positive deviation from the moving average, due to a peak in rainfall, into an increase in larval mortality. This parameter, so-called death factor, is introduced to represent the washout effect for the larvae. $\Theta\left(x\right)$ is x if x > 0 and 0 otherwise. Lastly, is a 12-month moving average calculated from the rainfall time series.

Variable d_L_ represents the larval development rate and is related to the ambient temperature following a simple regression used for *Anopheles gambiae*:

,

where T represents the temperature expressed in degrees Celsius.

The adult mosquito population is divided into three classes: susceptible non-infected mosquitoes (X), infected non-infectious mosquitoes, or becoming exposed when they bite an infectious human (V) and infectious mosquitoes (W). The system of ordinary differential equations is given by:

,

, and

.

Variable c denotes the human host to mosquito probability of transmission. Variables y and δ_M_ represent, respectively, the fraction of human population transmitting the parasite to the mosquito vector, and the adult mosquito mortality. The average lifetime of mosquitoes depends on temperature as follows:

.

Therefore, the per-capita death rate of adult mosquitoes can be written as: . γ_P_ is the per-capita rate at which new infectious mosquitoes arise, a quantity related to the duration of sporogony. This development time is also influenced by temperature as follows:

,

specifically for *Plasmodium falciparum* incubation within female mosquitoes.

The ABP model uses a simple regression between the inverse of the average gonotrophic cycle (), or roughly the biting rate (expressed in number of bites per mosquito per day), and the ambient temperature, as follows:

.

In the ABP full model version the human E class and the mosquito V class for the exposed individuals (not yet infectious) are divided into n_H_ and n_V_ latent classes (not shown here), respectively. The means of these incubation times are given by 1/γ_H_ and 1/γ_V_, respectively, whereas their variances decrease as function of the number of latent classes.

The outdoor ambient temperature drives the larval parameters δ_L_ and d_L_. The ABP model also considers an effective temperature, T_e_, which is higher than the outdoor ambient temperature, in the functions for adult mosquito mortality (δ_M_), biting rate (a), and the duration of sporogony (γ_P_). T_e_ is given by:

,

where x is the temperature weighting parameter, and $\Delta T$ is the maximum allowed difference between the maximum higher temperature adult mosquitoes can experience, T_i_, and the temperature outdoors, T_o_.

**Figure 1 Ross-Macdonald’s stock-flow model**

**Figure 2 Schematic diagram (top) and stock-flow diagrams of the SM3 and the SM6 sub-models (bottom) of the WCT mathematical tool**

**Figure 3 Stock-flow model of the human host component of the Alonso, Bouma and Pascual coupled mosquito-human malaria model**

**Figure 4 Stock-flow model of the mosquito population component of the Alonso, Bouma and Pascual coupled mosquito-human malaria model**
