# Supplementary material for: Testing a multi-malaria-model ensemble against 30 years of data in the Kenyan highlands
Source: Malar J. 2014 May 30;13:206. doi: 10.1186/1475-2875-13-206 (PMC4090176; doi:10.1186/1475-2875-13-206)
Supplement: Additional file 2 — Historical values and long-term trends in observed weather data. [file 1475-2875-13-206-S2.docx]

| **Climate variable [units]** | | | **Historical value** | **C.I. [95%]** | **Trend [units/decade]**  **α=0.05** |
| --- | --- | --- | --- | --- | --- |
| Rainfall (R) | R1 | Total annual R [mm] | 1,986.4 | 94.2 | N/S |
|  | R_DJF_ | Total DJF R [mm] | 291.3 | 60.5 | N/S |
|  | R_MAM_ | Total MAM R [mm] | 674.4 | 50.8 | N/S |
|  | R_JJA_ | Total JJA R [mm] | 510.7 | 45.1 | N/S |
|  | R_SON_ | Total SON R [mm] | 505.5 | 42.8 | N/S |
|  | R2 | Total number of dry days per year [number] | 140.4 | 6.3 | 7.4 |
|  | R2_DJF_ | Total dry days over DJF trimester [number] | 56.6 | 3.7 | N/S |
|  | R2_MAM_ | Total dry days over MAM trimester [number] | 28.6 | 2.3 | N/S |
|  | R2_JJA_ | Total dry days over JJA trimester [number] | 27.3 | 3.0 | N/S |
|  | R2_SON_ | Total dry days over SON trimester [number] | 27.9 | 2.7 | N/S |
|  | R3 | Maximum daily R [mm] | 35.4 | 1.9 | N/S |
|  | R4_DJF_ | Maximum dry spell over the DJF trimester [days] | 16.4 | 3.1 | N/S |
| Minimum temperature (Tmin) | MTmin | Annual Tmin on the warmest days [°C] | 13.9 | 0.2 | 0.4 |
|  | ATmin | Annual Tmin [°C] | 11.0 | 0.1 | 0.2 |
|  | mTmin2 | Annual Tmin on the coldest days [°C] | 7.9 | 0.1 | N/S |
|  | SDTmin | Day-to-day standard deviation of Tmin [°C] | 1.6 | 0.1 | 0.1 |
| Maximum temperature (Tmax) | MTmax | Annual Tmax on the warmest days [°C] | 26.5 | 0.1 | 0.2 |
|  | ATmax | Annual Tmax [°C] | 24.1 | 0.1 | 0.3 |
|  | mTmax2 | Annual Tmax on the coldest days [°C] | 20.8 | 0.2 | 0.2 |
|  | SDTmax | Day-to-day standard deviation of Tmax [°C] | 1.4 | 0.0 | N/S |
| Diurnal temperature range (DTR) | MDTR | Maximum annual DTR [°C] | 17.6 | 0.2 | N/S |
|  | ADTR | Annual DTR [°C] | 13.1 | 0.1 | N/S |
|  | mDTR2 | Minimum annual DTR [°C] | 7.9 | 0.2 | N/S |
|  | SDDTR | Day-to-day standard deviation of DTR [°C] | 2.4 | 0.1 | N/S |

C.I.: confidence interval; α: significance level; DJF: December-January-February; MAM: March-April-May; JJA: June-July-August; SON: September-October-November; N/S: non-significant
