# Supplementary material for: Testing a multi-malaria-model ensemble against 30 years of data in the Kenyan highlands
Source: Malar J. 2014 May 30;13:206. doi: 10.1186/1475-2875-13-206 (PMC4090176; doi:10.1186/1475-2875-13-206)
Supplement: Additional file 3 — Simulation outputs for base scenarios. [file 1475-2875-13-206-S3.docx]

**Simulation outputs for base scenarios**

Left panel: actual *Plasmodium falciparum* malaria incidence observed in Tea Plantation 1 over the period spanning from January, 1970 through October, 2004 (grey solid bars), along with malaria prevalence simulated by the MAC (black solid line labeled Sim prev – MAC), AM (green solid line labeled Sim prev – AM), WCT (blue solid line labeled Sim prev – WCT), and ABP (red solid line labeled Sim prev – ABP) models for the historical annual cycles of mean temperature and rainfall. The black thick line depicts the annual cycle of *P. falciparum* malaria prevalence obtained by averaging all models’ simulation outputs. The following parameter values were assumed for the MAC model: b=0.01; HD=20 days; WN=20 days; r=1/(HD+WN); α=exp(-1/1.229); υ=1.26 days; f_U_=36.5°C-days; l=5.0 °C; g_U_=9.9°C; U=υ + (f_U_/(T+l-g_U_)); p=α^(1/U); x_p_=0.01; T_e_=T+(1-x_p_)*l; a=0.091678*T_e_-1.7982; and d=27,000 individuals. For the AM model (besides the set of parameters proposed for the MAC model): f_N_=116°C-days; g_N_=16°C; n=f_N_/(T+l-g_N_); t_h_=13 days; t_m_=10 days; μ_1_=(1/(70*365)) days^-1^; μ_2_=(1/21) days^-1^; and c=1.0. For the WCT model (besides the set of parameters proposed for the MAC and AM models): μ=6,500; C=0.24; β=exp(-1/0.4); x=0.1; h=0.8; k=1; v=0.4; r_WCT_=1/(80/30) month^-1^; and λ=1.00. And for the ABP model (besides the set of parameters proposed for the MAC, AM and WCT models): δ_H_=1/20*(1/135); b_ABP_=0.5; β_e_=0.0000257; σ_0_=0.0805; ρ=0.428; r_0_=0.00555; γ=0.0486; ξ=0.825; η=0.0346; ν=0.05; F=66; k_A_=695; k_E_=0.198; δ_0_=0.0329; c_ABP_=0.365; x_ABP_=0.01; and ΔT=4.2°C. Right panel: actual *P. falciparum* malaria incidence (grey solid bars) along with malaria prevalence simulated by the WCT model for various mean durations of infectivity, 1/r_WCT_ (from 40 to 95 days).
