# Supplementary material for: Testing a multi-malaria-model ensemble against 30 years of data in the Kenyan highlands
Source: Malar J. 2014 May 30;13:206. doi: 10.1186/1475-2875-13-206 (PMC4090176; doi:10.1186/1475-2875-13-206)
Supplement: Additional file 4 — Individual model simulation outputs [50-66]. [file 1475-2875-13-206-S4.docx]

(A)

(B)

(C)

(D)

**Individual model simulation outputs**

Monthly *Plasmodium falciparum* malaria incidence observed in Kericho over the period spanning from January, 1979 through October, 2004 (grey solid bars), along with the 25%, 50% and 95% percentiles of the distributions of monthly *P. falciparum* malaria prevalence simulated by the MAC (panel A), AM (panel B), WCT (panel C), and ABP (panel D) models, for the actual climatic conditions, for the period spanning from January, 1979 through December, 2009, and for 1-, 1-, 2-, and 0-month time lags, respectively. The following parameter ranges were assumed for the MAC model: b=[0.001;0.010] (10 values); HD=[11;26 days] (9 values); WN=[12;30 days] (10 values); α=[0.4;0.6] (11 values); υ=[0.5;4.0 days] (9 values); f_U_=[36.5;37.1 °C-days] (7 values); l=[1;10 °C] (10 values); g_U_=[7.7;9.9 °C] (12 values); x_p_=[0.00;1.00] (12 values); and d=[fixed constant]. For the AM model (besides the set of parameters proposed for the MAC model): f_N_=[111.0;204.4 °C-days] (11 values); g_N_=[14.2;19.0 °C] (11 values); t_h_=[5.5;16 days] (12 values); t_m_=[8;30 days] (8 values); μ_1_=[(1/(60*365));(1/(80*365)) days^-1^] (5 values); μ_2_=[(1/30);(1/11.2) days^-1^] (5 values); and c=[unknown; fixed constant]. For the WCT model (besides the set of parameters proposed for the MAC and AM models): μ=[2,000;10,000] (10 values); C=[0.00;0.50] (7 values); β=[unknown; fixed constant]; x=[0.0;0.3] (7 values); h=[0.5;1.0] (6 values); k=[0.0;1.0] (10 values); v=[0.0;1.0] (10 values); r_WCT_=[1/(666.66/30);1/(25.97/30) month^-1^] (10 values); and λ=[unknown; fixed constant]. And for the ABP model (besides the set of parameters proposed for the MAC, AM and WCT models): δ_H_=[fixed constant]; b_ABP_=[0.01;0.50] (6 values); β_e_=[0;10^-4^] (12 values); σ_0_=[0.00;0.10] (12 values); ρ=[0.00;1.00] (11 values); r_0_=[0.00;0.01] (12 values); γ=[0.0475;0.1435] (11 values); ξ=[fixed constant]; η=[0.00;0.10] (12 values); ν=[0.2;1.0] (10 values); F=[fixed constant]; k_A_=[0;3*103] (8 values); k_E_=[0;0.3] (7 values); δ_0_=[fixed constant]; c_ABP_=[0.00;0.379] (9 values); x_ABP_=[0;1] (12 values); and ΔT=[2.2;5.0°C] (8 values). Literature ranges are reported in 36 and [50-66].
